# Undergraduate Public Health Education: Is There an Ideal Curriculum?

**DOI:** 10.3389/fpubh.2015.00016

**Published:** 2015-02-02

**Authors:** Leonard H. Friedman, Joel M. Lee

**Affiliations:** ^1^Milken Institute School of Public Health, The George Washington University, Washington, DC, USA; ^2^John A. Drew Professor of Healthcare Administration, The University of Georgia College of Public Health, Athens, GA, USA

**Keywords:** undergraduate students, undergraduate public health education, public health, curriculum, education, medical, competencies, BSPH

This commentary speaks to the need to design a curriculum that best meets the needs of students who are enrolled in Bachelor of Science in Public Health (BSPH) degree programs. The past 10 years has witnessed a dramatic growth in the number and size of these programs. While several of these degrees are housed within schools and colleges of public health, many are located in colleges of arts and science, allied health, medicine, and many other academic homes. It is difficult to determine the actual number of BSPH programs (or similar type degrees but with different names) due to a lack of national accrediting standard. The number of students in these programs varies widely with as few as 25 to as many as 600. In addition to stand-alone BSPH degrees, there are a number of what is known in the field as three plus two programs where a student spends 3 years completing their undergraduate courses and then an additional 2 years of study that completes both their BS degree in addition to their Master of Public Health (MPH).

The point here is that there is a significant demand for the BSPH degree on the part of students and universities are more than happy to meet that demand. A legitimate question that we have is what career or education paths do students pursue once they complete their BSPH degrees. Evidence suggests that the largest fraction of BSPH students use the degree as pre-professional preparation for entry into advanced clinical training including medicine, nursing, physical therapy, physician assistant, pharmacy, and other specialty areas. Given the implementation of the Affordable Care Act and the movement toward population health, it is critical that future clinicians know and can potentially apply core public health principles into their practices.

A second path for BSPH graduates is into MPH degree programs. Once the spark is ignited during their undergraduate education, students see that they can make a profound difference in public health practice but know that the MPH is the degree of choice for a large number of employers. In our companion commentary (“Undergraduate Public Health, Lessons Learned from Undergraduate Health Administration Education”), we ask whether courses taken in the BSPH degree might have the capacity to either transfer into the MPH or perhaps students should be waived out of one or more of the core courses and instead be allowed to take additional electives. If this practice were to become commonplace, prospective MPH students might see an opportunity to better tailor their graduate education.

In addition to preparation for clinical education or entry into an MPH program, current BSPH students sometimes move directly into Public Health related jobs at the entry level where they obtain important work experience before advancing to an MPH degree. There are also students who upon graduation go into a whole variety of other education or work related opportunities including Peace Corps or Teach for America. Given the four paths that BSPH graduates can potentially take, it is important to clearly understand where students go once they depart our programs and how our curricula can add value to the career options for graduates. Toward that end, does it make sense to craft a single unified BSPH curriculum that provides a rigorous preparation in public health and at the same time is flexible enough to accommodate the multiple career paths of graduates? Although various frameworks have been proposed (AAC&U, ASPPH, and CEPH), no model has been developed in sufficient detail to consider the challenges of implementing a truly well-articulated program. Do we as a field wish to develop a BSPH curriculum that mirrors the current CEPH requirement for accredited MPH degrees with a required core and multiple program tracks? If this is the option selected, what differentiates the BSPH from the MPH? As noted in our companion commentary, graduates from AUPHA certified undergraduate health administration programs compete successfully for entry level management jobs with graduates from CAHME accredited MHA programs. Regardless of how we design our BSPH curriculum, programs need to keep in mind what is in the best interest of their students.

As programs think about either designing a BSPH curriculum *de novo* or modifying an existing curriculum, there are four important elements to keep in mind:

- Begin with a market analysis of alumni and prospective employers. It is vital for BSPH programs to have a good handle on where graduates are going and then determining how well they are preparing students to enter the market. One commonly used strategy is to regularly survey alumni to determine where their first jobs take them.
- What are the competencies that are needed for the commonly taken career paths of alumni? Public health education has been steadily moving toward providing students with competencies that address what they can do rather than just what they know. In this case, programs will need to speak with the employers of graduates to get a well-defined sense of required competencies. A good approach here is to create an external advisory committee made up of practitioners and alumni to advise program faculty on the most appropriate competencies.
- What are your unique programmatic strengths and how can those strengths create market opportunities for graduates? Given the sorts of resource constraints that are part of virtually every college and university, programs cannot afford to be everything to everyone. Choose a few areas that are particularly strong and build a curriculum around those. One thing a program might do is a SWOT analysis in which an assessment of programmatic strengths, weaknesses, opportunities, and threats is performed. Given that information the program faculty are better equipped to create a market sensitive curriculum.
- Michael Porter at the Harvard Business School talks about creating value as a way of standing out in a competitive environment. How do programs bring value to students and why should they enroll in your program rather than your local or national competitors? What brand recognition does your university or program possess? For potential employers of graduates, why should they hire your students rather than from competing BSPH programs or undifferentiated BA/BS graduates? What sorts of activities can be done that helps to allow your program to stand out?

Once the program has gone through the hard work of looking at the market for graduates and the forces exerted by competitors, it is time to think about the nuts and bolts of the curriculum. In general, the authors recommend the following five criteria – all of which are influenced by your global university undergraduate requirements and the outcome of your market analysis, competency decisions, programmatic strengths/weaknesses, and value determination.

- University general education requirements – whether called the GE, Baccalaureate Core, or some other name, virtually all BA/BS students need to complete these classes (typically) before their upper division courses begin. One good way of recruiting new students is to make sure that one or more public health courses are part of the general education requirement.
- Common Public Health classes – sometimes referred to as the public health core, these are the classes that all BSPH students are required to complete early in their career. Given that most schools require students to declare a major by the start of their junior (third) year, it is recommended that this common core be made available for second and third year students.
- Required domain specific classes – at this point, important decisions need to be made. If the program decides to offer a couple of common public health domains (e.g., health promotion, environmental health, and health policy) it is then up to the faculty to determine which classes are needed to fulfill the requirements for each of the domains. Alternatively, the decision might be made to offer a generalist BSPH that does not divide into distinct domains. In this case, the program will still want to make available a set of required Public Health classes beyond the common core.
- Electives – the authors strongly recommend that an opportunity be provided for BSPH students to take a number of elective credits to enhance their depth of understanding of public health domains of particular interest.
- Field experience – it is essential that all BSPH students be given the opportunity to apply what they have learned in the classroom to a real-time field experience. While many schools are embracing service learning as part of the classroom experience, this is (in our estimation) not adequate. An ideal field experience would be a semester/quarter long. The field experience (or some other name) should be supervised by an experienced public health practitioner and would be a required, credit bearing experience.

In addition to the outline that has been detailed here, there are a number of other important attributes to a highly effective BSPH curriculum. Students need the opportunity to develop their skills as professionals. What are the professional norms in the field and how are these transmitted to students? While some of these professional norms will be organization dependent, others are cross-cutting across public health including respect for others, dignity, enhancing diversity, and building cultural competence. For those of us in faculty roles, let us never forget that we too serve as role models for our students and if we want our students to behave in a professional manner, we need to do the same thing.

Leadership is another critical skill for BSPH students to develop. Leadership is not confined to persons holding executive management positions and should be part of the educational preparation of all public health students. It is not enough to attend a lecture about leadership or to read the latest leadership book. Students must get out and practice being a leader – and along with this learn what it is like to fail. Student led clubs and organizations are an ideal way for students to begin to hone their skill and talent in leadership. Faculty mentoring will be a critical part of any student led activity.

Public health is a team sport and BSPH students need to be given the opportunity to work in teams throughout the curriculum. Most students dread this experience but it is vital that they learn how to work effectively with diverse team members who possess differential skills and varying levels of motivation and commitment. Through group work, perhaps most importantly, students learn how to deal with group conflict.

Finally, identify alumni and other local public health leaders who are willing to come in and talk with students about the breadth of opportunities in the field and provide an insider’s perspective on their work. Sometimes called Executives in Residence (EIR’s), these people can help with mock interviews, resume reviews, and can serve as mentors to current students.

In conclusion, the authors are “bullish” on the future of the BSPH degree. We believe that the demand for this degree will only get stronger in the years to come. Given this level of confidence, it is important to recognize that your students are pursuing multiple pathways after graduation. In order to be most closely aligned with the needs of the market, you will need to develop, implement, and continuously evaluate a set of competencies for program graduates. Along with the competencies, answer the value proposition question – why should students study with you and why should employers hire your graduates? In order to do this, you can craft a curriculum that simultaneously provides flexibility, rigor, and practical value to your students.

## Conflict of Interest Statement

The authors declare that the research was conducted in the absence of any commercial or financial relationships that could be construed as a potential conflict of interest.

## Author Note

The authors who presently serve in administrative leadership positions in graduate education have both previously served as directors of undergraduate programs in health administration as well as on committees and the Board of the Association of University Programs in Health Administration and as site visitors for Council on Accreditation in Health Management Education.

